# Maternal diabetes negatively impacts fetal health

**DOI:** 10.1098/rsob.220135

**Published:** 2022-09-21

**Authors:** Cecilia González Corona, Ronald J. Parchem

**Affiliations:** ^1^ Center for Cell and Gene Therapy, Stem Cells and Regenerative Medicine Center, One Baylor Plaza, Houston, TX 77030, USA; ^2^ Molecular and Cellular Biology, Baylor College of Medicine, Houston, TX, USA

**Keywords:** diabetes, maternal–fetal health, birth defects, hyperglycaemia, pancreas, insulin

## Abstract

Diabetes is a chronic metabolic disease affecting an increasing number of people. Although diabetes has negative health outcomes for diagnosed individuals, a population at particular risk are pregnant women, as diabetes impacts not only a pregnant woman's health but that of her child. In this review, we cover the current knowledge and unanswered questions on diabetes affecting an expectant mother, focusing on maternal and fetal outcomes.

## Graphical abstract

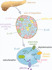

## 1. Introduction

Diabetes mellitus is a chronic metabolic disease affecting the production of insulin by pancreatic β cells. Insulin is a negative regulator of glucose; without it, blood glucose levels rise to life-threatening levels [1,2]. Worldwide rates of diabetes have only been increasing: 171 million adults were estimated to be diabetic in 2000, with the number jumping to 536 million by 2021 [3,4]. By 2045, the number of diabetic adults is predicted to be more than 783 million [4]. In 2019, diabetes was the ninth leading global cause of death, and it is among the top 20 conditions causing disability worldwide [5,6]. Of the various subtypes of diabetes, of importance to this review are type 1 diabetes mellitus, (T1DM), type 2 diabetes mellitus (T2DM) and gestational diabetes mellitus (GDM).

While T1DM and T2DM irrefutably cause embryopathies, or defects in the developing embryo, whether GDM does as well remains controversial for its later screening. However, the percentage of pregnant women with pre-existing T1DM or T2DM, which we differentiate from GDM and deem maternal diabetes, is rising [7–9]. Maternal diabetes poses a risk to a mother's present and future health while putting her child at risk for several birth defects or later health issues, including the development of diabetes [10–14]. Thus, the diabetic cycle begets itself.

The current treatments for diabetes involve artificial insulin and blood glucose regulation by oral or intravenous medications [15]. The prescribed drug varies by the type of diabetes diagnosed, though insulin is the most common treatment [16]. However, all drugs used to treat diabetes can have significant side effects (table 1). Some pharmacological agents, such as insulin and metformin, are approved for use during pregnancy, but most oral hypoglycaemic agents used in non-pregnant patients have unknown short- and long-term effects on both the mother and fetus [24]. Diabetes management and treatment is also onerous, requiring the individual to carefully monitor their blood glucose levels and consequent insulin administration every day for the rest of their lives. Moreover, the cost of lifelong treatment adds up. In the USA, the annual cost of diagnosing and treating diabetes and prediabetes is more than USD300 billion [25]. In fact, insulin costs in the USA—where its cost is not nationally capped—are four times higher than the country with the next most expensive insulin, and it is 10 times more expensive than the global average price of insulin [26]. Insulin analogues have been developed in recent years, and these too are becoming more expensive, despite no clear evidence that they perform better than the standard synthetic human insulin [27].

**Table 1.** Drugs used for the treatment of diabetes.

drug	drug class	mechanism	approved in pregnant women?	side effects	source
insulin/insulin analogues (i.e. insulin aspart, lispro, glulisine, insulin glargine, detemir and degludec)	protein hormone	directly interacts with pancreatic β cells and increases cellular uptake of glucose	yes (except for degludec)	hypoglycaemia, weight gain, insulin hypersensitivity, insulin resistance, lipoatrophy, lypohypertrophy	Cheung [17]; Sabetsky & Eblom [16]; Doder *et al*. [18]; Koren *et al*. [19]; Donner & Sarkar; [20]; Sprio *et al*. [21]; Jethwani *et al*. [22]
metformin	biguanide	increases glucose utilization and lactate production in the gut; increases insulin signalling in the liver	yes	abdominal discomfort, gastroenteritis, metformin intolerance	Tahrani *et al*. [23]; Cesta *et al*. [24]
tolbutamide, chlorpropamide, glibenclamide (glyburide), gliclazide, glipizide, glimepiride	sulfonylurea	increases insulin exocytosis	only glibenclamide (glyburide)	pancreatic β cell desensitization, worsening hepatic and/or renal impairment, impairment of sulfonylureas, hypoglycaemia, weight gain	Tahrani *et al*. [23]; Cesta *et al*. [24]
nateglinide, repaglinide	meglitinide/ glinides	increases insulin release	no	hypoglycaemia, weight gain	Tahrani *et al*. [23]
acarbose, migitol, voglibose	α-glucosidase inhibitor	reduces blood-glucose interactions, thereby reducing prandial insulin levels	acarbose has been tested in pregnant women, but data is limited	gastroenteritis, hypoglycaemia	Cheung [17]; Tahrani *et al*. [23]
pioglitazone, rosiglitazone, troglitazone	thiazolidinedione	promotes insulin sensitivity and glucose uptake; reduces gluconeogenesis in the liver and lowers free fatty acid levels, thus improving β cell viability and glucose utilization	no	liver damage, cardiovascular complications, bladder cancer, edema, weight gain, osteoporosis	Tahrani *et al*. [23]
sitagliptin, vildagliptin, saxagliptin, linagliptin, alogliptin, omarigliptin, trelagliptin	DPP-4 inhibitor	improves insulin secretion and β cell proliferation; decreases glucagon secretion	no	gastroenteritis, hypoglycaemia, acute pancreatitis	Tahrani *et al*. [23]
exenatide, liraglutide, lixisenatide, albiglutide, dulaglutide	GLP-1RA	improves insulin secretion, decreases glucagon secretion	no	nausea, hypoglycaemia, injection-site reactions	Tahrani *et al*. [23]
dapagliflozin, canagliflozin, empagliflozin	SGLT2 inhibitor	increases glucose elimination via urine	no	hypoglycaemia, genital and urinary tract infections, hypercholesteraemia, diabetic ketoacidosis	Tahrani *et al*. [23]

With increasing diabetes diagnoses, its health and economic costs will only grow. It is pertinent to study diabetes and determine safer and more cost-effective treatment options and, more importantly, modes of prevention. However, as there are various types of diabetes, each differing in its aetiology, treatment and manifestations in men and women, studies must carefully consider their approach. In this review, we cover the current knowledge and unanswered questions on diabetes affecting an expectant mother, focusing on maternal and fetal outcomes.

## 2. Types of diabetes

Before the development of overt diabetes, patients can develop impaired glucose tolerance, termed prediabetes. Prediabetic individuals have blood glucose levels above the homeostatic threshold but not high enough to be diagnosed as diabetes [28]. In 2018, the USA had 88 million people aged greater than or equal to 18 years who were prediabetic [29]. Classically, diabetes was categorized into juvenile diabetes and adult diabetes, with disease onset observed by the eponymous age [30]. These classifications have changed as more has been learned about diabetes, and the main types are considered T1DM, T2DM and GDM [30]. An estimated 34.1 million adults in the USA are diabetic, although this number excluded those affected by GDM [29]. The American Diabetes Association recognizes other subtypes of diabetes, but their causes are quite rare and specific—such as by cystic fibrosis, organ transplantation and monogenic defects—and these subtypes will not be covered in this review. For more information on these other types of diabetes, the reader is referred to the American Diabetes Association's *Classification and Diagnosis of Diabetes: Standards of Medical Care in Diabetes—2019*.

T1DM is characterized by the complete or near-complete absence of insulin due to the destruction of pancreatic β cells, the cells responsible for insulin production [31]. T1DM is differentiated from T2DM in that it is an autoimmune disease, meaning the body destroys its own β cells; but the result is the same loss of insulin production leading to high blood glucose levels, or hyperglycaemia, and eventually diabetes. As insulin-producing β cells are not viable in someone with T1DM, they must take insulin or its analogue to survive.

By contrast, T2DM is primarily caused by dysfunctional β cells [32]. In an affected individual, β cells will not properly detect high blood glucose levels, and thus cannot be stimulated to release the correct amounts of insulin to trigger the uptake of blood glucose into the cells of various organs [32]. Eventually, higher compensatory levels of insulin must be released to lower blood glucose, which drives insulin resistance [33]. The continued cycle of increased blood glucose levels requiring increased insulin levels for abatement results in T2DM [32]. Unlike T1DM, the loss of insulin in T2DM is relative and not absolute [34]. Studies tend to group the prevalence of T1DM and T2DM, but 90–95% of diabetics in the USA are estimated to have T2DM [29].

Lastly, GDM occurs when a pregnant woman is diagnosed with diabetes in her second to third trimester that is not clearly pre-existing T1DM or T2DM [30]. Screening for GDM is recommended between 24 and 28 weeks of gestation unless a woman is at high risk of developing it, in which case screening should be carried out at the first prenatal visit [35]. Treatment can begin if GDM is diagnosed, as returning blood glucose levels to normalcy is paramount in decreasing the risk for morbidity and mortality in the child [35]. There is no global standard for diagnosing GDM, as GDM can be difficult to distinguish from diabetes not induced by pregnancy—that is, diabetes that would have still been diagnosed if an expectant mother was not pregnant [36]. The World Health Organization recommends the criteria set forth by the International Association of Diabetes in Pregnancy Study Groups, in which GDM is diagnosed if hyperglycaemia is detected during pregnancy but is below what would be considered diabetic blood glucose levels outside of pregnancy [37]. The prevalence of GDM in the USA is 7.6%, with 19.6% of cases later developing into T2DM [38].

In this review, we differentiate between GDM and maternal diabetes, which we deem pre-existing diabetes in a pregnant woman, or, per the World Health Organization, ‘diabetes in pregnancy’.

## 3. Diabetes diagnosis

Diabetes can be diagnosed in multiple ways, but in all except T1DM, hyperglycaemia detection is key (table 2). Because T1DM is an autoimmune disease, it is detected by the presence of antibodies against β cell antigens, which arise before clinical symptoms are observed [42]. There are various tests for detecting T2DM. The most reliable yet most expensive T2DM diagnostic test is for haemoglobin A1c [41]. Glucose binds to haemoglobin within red blood cells and this glycated haemoglobin, termed HbA1c, can be measured as a percentage of total haemoglobin. It is then used as a readout of the average blood glucose level over the last 2–3 months, which is the lifespan of a red blood cell [40]. In the fasting plasma glucose test, an individual's blood glucose is measured after at least an 8 h fast, typically overnight [30]. In the oral glucose tolerance test, a 75 g sugary drink is ingested following an overnight fast, and the body's processing of the sugar is tracked [30]. Modified versions of this test are used for diabetes screening in pregnant women: an overnight fast and consumption of a 75 g sugary drink may be done. Alternatively, a non-fasted woman can take a 50 g sugary drink followed by a fast and a 100 g sugary drink [30]. The least reliable test, but the least onerous for the patient, is the random plasma glucose test. As the name implies, blood glucose is measured without preparation and no fasting is needed [41].

**Table 2.** Diagnostic tests for T1DM, T2DM and GDM. Note the variability in the oral glucose tolerance, two-step criteria for GDM. The first set of numbers corresponds to Carpenter–Coustan's diagnostics and the second set to the National Diabetes Data Group's. Both are approved by the American Diabetes Association.

test	subtype diagnosed	mechanism	levels	source
antibody test against β cell antigens	type 1	detects antibodies against GAD65 (glutamic acid decarboxylase), IA-2 (tyrosine phosphatase-like protein), IAA (insulin), ZnT8 (zinc transporter)	considered diabetic if two or more antibodies are detected	Bonifacio [39]
haemoglobin (Hb) A1c	type 2	measures glycated haemoglobin, which acts as a proxy for average blood glucose levels over the past three months	diabetic: 6.5%	Eyth & Naik [40]
			prediabetic: 5.7–6.4%	
			non-diabetic: <5.7%	
fasting plasma glucose	type 2	measures blood glucose after ≥8 h fasting	diabetic: ≥126 mg dl^−1^	American Diabetes Association [30]
			prediabetic: 100–125 mg dl^−1^	
			Non-diabetic: <100 mg dl^−1^	
oral glucose tolerance	type 2	measures the body's response to a glucose bolus	diabetic: ≥200 mg dl^−1^	American Diabetes Association [30]
			prediabetic: 140–199 mg dl^−1^	
			non-diabetic: <140 mg dl^−1^	
random plasma glucose	type 2	measures blood glucose non-fasting at random time	diabetic: ≥200 mg dl^−1^	American Diabetes Association [30]
			prediabetic: 140–199 mg dl^−1^	
			non-diabetic: <140 mg dl^−1^	
oral glucose tolerance, one-step	gestational	sugar processing	diabetic: ≥92 mg dl^−1^ after fasting, ≥180 mg dl^−1^ after 1 h, ≥153 mg dl^−1^ after 2 h	American Diabetes Association [30]
oral glucose tolerance, two-step	gestational	sugar processing	first step, diabetic: ≥130 mg dl^−1^	Genuth *et al*. [41]
			second step, diabetic: ≥95 or 105 mg dl^−1^ after fasting, ≥180 or 190 mg dl^−1^ after 1 h, ≥155 or 165 mg dl^−1^ after 2 h, ≥140 or 145 mg dl^−1^ after 3 h	

## 4. General pathophysiology of diabetes

**Figure 1.**
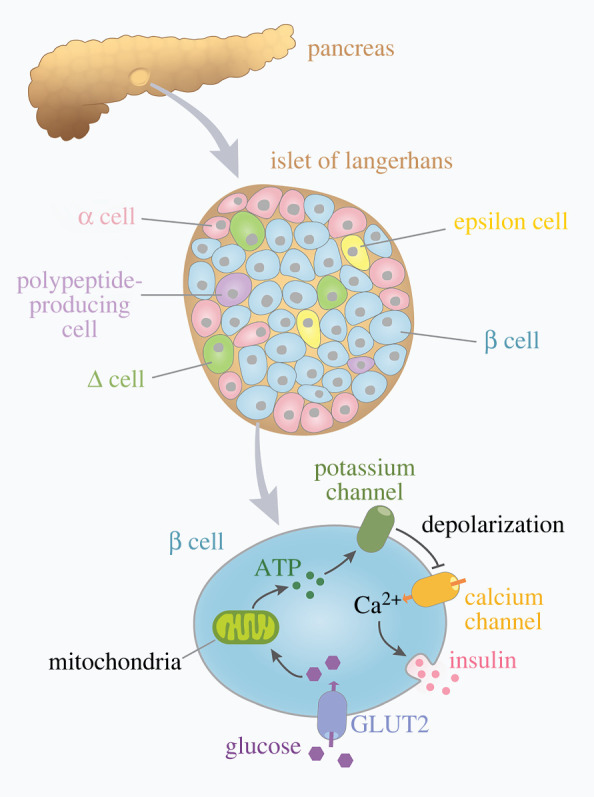
Overview of insulin release in the pancreatic beta cell. The pancreas is partly composed of islets of Langerhans. Like the name suggests, the islets are island-shaped structures made up of numerous cell types: α cells, β cells, delta cells, epsilon cells and polypeptide-producing cells. In β cells, glucose uptake is mediated by the GLUT2 transporter. Following glucose metabolism in the mitochondria, ATP is released, stimulating potassium channels to close. This depolarization triggers voltage-gated calcium channels to open. As calcium enters the cell, insulin is exocytosed. In a diabetic person, insulin is either not produced (i.e. T1DM) or is not released at appropriate levels (i.e. T2DM and GDM), but in all types of diabetes, blood glucose is unable to be regulated and thus rises.

**Figure 2.**
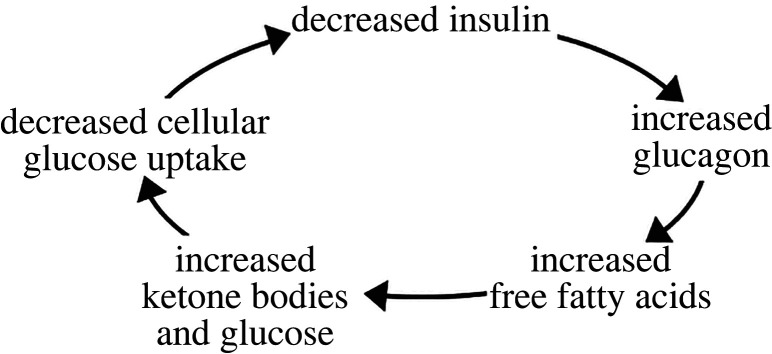
Overview of the metabolic effects of diabetes. A person with diabetes has low levels of insulin and high levels of glucagon. This stimulates release of free fatty acids, which act as the substrate for formation of ketone bodies. Free fatty acids decrease the capacity of a cell to take up glucose, while excessive ketones lead to ketoacidosis. Glucose is simultaneously overproduced. The high presence of glucose and its inability to be regulated due to low insulin worsens the metabolism of a diabetic individual.

Insulin is produced by the pancreas, specifically by β cells. β cells are arranged within islets of Langerhans, together with α cells, which produce glucagon; Δ cells, which produce somatostatin; epsilon cells, which produce ghrelin; and polypeptide-producing cells (figure 1). Insulin is a negative regulator of blood glucose, the levels of which must be maintained within a specific range for an individual to be in good health [1,2]. Insulin is secreted in response to eating, as glucose is primarily derived from the diet [43]. In a non-diabetic individual, the presence of high glucose stimulates β cells to release insulin, lowering blood glucose levels [34]. Other nutrients, hormones and neurotransmitters activate insulin secretion, but in terms of diabetes, glucose is the most important chemical factor [44]. In a diabetic individual, a cascade of metabolic dysfunction occurs in the presence of high glucose. Because glucose is a diuretic, diabetic individuals often suffer from an osmotic imbalance leading to fluid loss and dehydration [45]. Lack of insulin also leads to an increase in glucagon, a hormone acting in opposition to insulin that typically induces the liver to produce ketone bodies [46,47]. In turn, the increase in glucagon can cause diabetic ketoacidosis, characterized by hyperglycaemia, metabolic acidosis, high levels of ketones in the blood or urine, and often a decreased circulatory fluid volume [48]. Diabetic ketoacidosis is also more common in T1DM than in T2DM [48]. Insulin typically inhibits formation of ketone bodies while glucagon promotes it via catabolism of fatty acids. Thus, in diabetes, with low levels of insulin but high levels of glucagon, the increased release of fatty acids forms ketone bodies [45,49]. Fatty acid accumulation prevents glucose uptake in muscle cells and further worsens the metabolic state of the individual with diabetes [50]. The cycle is summarized in figure 2.

### 4.1. Physiology of T1DM

In T1DM, the pancreas fails to produce insulin due to autoimmune β cell destruction [10,51]. The exact reasons for this are unknown, but genetic predisposition in combination with environmental cues is likely to be responsible. A leading hypothesis is that recruited cytotoxic T cells and macrophages secrete cytokines, free radicals, and nitric oxide, all capable of killing β cells [52–55]. When greater than or equal to 80% of β cells are destroyed, clinical symptoms of T1DM typically appear [56]. These symptoms include polydipsia (excessive thirst), polyuria (excessive urination), polyphagia (excessive hunger), extreme fatigue, blurry vision, slow wound healing, very dry skin, tingling in the extremities and unexplained weight loss [57].

### 4.2. Physiology of T2DM

In contrast to T1DM, the hyperglycaemia seen in individuals with T2DM is driven largely by insulin resistance. Insulin resistance occurs when normal concentrations of insulin do not produce the adequate biological response of cellular glucose uptake. This further lowers insulin sensitivity, as a greater concentration of insulin is required to achieve blood glucose homeostasis [58]. In contrast to the autoimmunity-induced β cell apoptosis seen in T1DM, β cell dysfunction and death in T2DM are caused by chronic exposure to high glucose (hyperglycaemia) and high free fatty acids (hyperlipidaemia), as well as an accumulation of human islet amyloid polypeptide (hIAPP) [59,60]. Exactly how hyperglycaemia and hyperlipidaemia induce β cell apoptosis is not fully understood, but prolonged high blood glucose levels could place stress on the endoplasmic reticulum, passing a threshold that induces apoptosis [61]. Hyperglycaemia also leads to high levels of reactive oxygen species, which are lethal to β cells, and to high levels of cytoplasmic calcium, which is potentially pro-apoptotic [62,63]. When hIAPP, which is co-secreted with insulin, misfolds and accumulates in the space between β cells and islet capillaries, it increases the likelihood of hyperglycaemia while also occurring alongside it [60,64]. How misfolded and accumulated hIAPP kills β cells remains to be elucidated, but proposed mechanisms include oxidative stress, endoplasmic reticulum stress, mitochondrial stress, prevention of autophagy, and a change in the cell membrane permeability [64]. To compensate for β cell loss, extant β cells will increase in mass, but over time this ability is exhausted [51,56]. It typically takes 9–12 years for β cells to degrade enough that an individual can be diagnosed with T2DM [65]. Similar to T1DM, clinical symptoms of T2DM are polydipsia (excessive thirst), polyuria (excessive urination), polyphagia (excessive hunger), extreme fatigue, blurry vision, slow wound healing, very dry skin, tingling in the extremities and unexplained weight loss [66].

### 4.3. Physiology of GDM

During a non-diabetic pregnancy, the liver produces greater amounts of glucose, but fasting blood glucose levels fall as gestation progresses, most likely due to increased blood plasma volume or increased glucose utilization by the fetus or mother [67]. Fasting insulin levels also increase, but as glucose production rises rather than falls, insulin sensitivity decreases [67]. In obese and lean women with GDM compared to weight-matched non-diabetic pregnant women, fasted blood glucose and insulin levels are high but hepatic glucose production is unchanged, suggesting impaired β cell function [68,69]. Women with GDM also have high free fatty acid levels due to a decrease in free fatty acid suppression by insulin [67]. Insulin sensitivity and dysfunctional β cells may be present before pregnancy but are detected during pregnancy due to common prenatal metabolic screening [36]. The mechanisms by which GDM-affected women become less insulin and lipid sensitive are not well understood, but studies have shown that downstream insulin effectors, insulin receptor substrates 1 and 2 (IRS-1 and IRS-2) decrease in GDM, whereas P85, a subunit of phosphatidylinositol-3-kinase (PI3-K), is increased [70]. Furthermore, women with GDM have a deficiency in glucose transporter-4 (GLUT-4), which is sensitive to insulin, and the insulin receptor part not expressed on the cell surface has a decreased ability to be phosphorylated by tyrosine [67,71]. This impaired phosphorylation is likely compounded by the increase in plasma cell membrane glycoprotein-1, an inhibitor of the insulin receptor tyrosine kinase (IRTK) that phosphorylates IRS-1 [67]. Women with GDM also have increased levels of tumour necrosis factor-α, which inhibits IRS-1 and IRTK [72]. Most women with GDM will not have clinical symptoms, but in those who do, they may include increased thirst and more frequent urination [73].

## 5. Risk factors

### 5.1. Risk factors for T1DM

Individuals born to a mother aged greater than or equal to 35 years with maternal obesity prior to or during early gestation are at an increased risk of developing T1DM [74–76]. Family history plays a role, as a child born to a mother with T1DM has a 1 in.40 chance of developing the same disease; this jumps to 1 in.15 if the father has T1DM, likely for epigenetic reasons [77].

Genetically, variations in the human leucocyte antigen (HLA) gene are also a risk factor for T1DM [78]. In humans, *HLA* encodes for genes in the major histocompatibility complex, which is required for recognizing foreign agents in the body and mounting an appropriate immune response [79]. Genes associated with a high childhood body mass index (BMI) have also been implicated in T1DM [80].

Beginning in the 1950 s, there has been a global increase in childhood T1DM diagnoses, even when accounting for genetic factors. Thus, the role of the environment in developing T1DM cannot be ignored [10]. However, upon *in silico* analysis, no single environmental factor could explain the sudden and dramatic increase in T1DM [10]. An unhealthy diet could perhaps contribute to the trend, along with genetic factors. High sugar intake is known to lead to T1DM progression but does not cause β cell autoimmunity [81]. High consumption of cow milk products in early childhood has been found to increase the risk of islet autoimmunity and progression to T1DM [82,83]. In animal studies, a gluten-free or fibre-rich diet has been found to reduce risk of T1DM, but comparative studies in humans have been inconclusive [84–87].

Viral infections may also contribute to β cell destruction, with several serotypes of the enterovirus Coxsackievirus B being the prime suspects, though findings from meta-analyses have varied [88,89].

Importantly, because T1DM typically has an earlier onset than T2DM, the risk factors have been studied mostly in those less than 20 years of age, but more adults than children are increasingly diagnosed with T1DM [90].

### 5.2. Risk factors for T2DM

Because diabetes is characterized by elevated blood glucose and glucose is obtained via the diet, a major risk factor in developing T2DM is an unhealthy diet, i.e. a diet high in processed meat, red meat, fried foods, refined grains, and sugar [91,92]. Such a diet can lead to obesity, an additional risk factor for T2DM [91,92]. Other unhealthy activities, such as smoking and lack of exercise, are also strongly associated with T2DM [91].

The diet described above is prevalent in Western countries [92]. It is also spreading into developing countries, where the prevalence of T2DM is increasing faster than in developed countries [93,94]. Poverty is also associated with a higher risk of T2DM, but that an individual with low socioeconomic status is more likely to follow the inexpensive Western-style diet and lead a sedentary lifestyle cannot be overlooked [91,95].

Biological factors can also influence the onset of T2DM. High levels of serum biomarkers, such as alanine aminotransferase, γ-glutamyl transferase, C-reactive protein, and uric acid, are associated with T2DM [91]. These are markers of inflammation, which are linked to β cell dysfunction [91]. Genes identified as T2DM risk factors are *CAPN10,* which encodes calpain-10, a ubiquitously expressed cysteine protease; *TCF7L2,* encoding transcription factor-7 like 2, a transcription factor involved in blood glucose homeostasis; *PPARG,* encoding peroxisomal proliferative activated receptor γ (PPAR*γ*), a regulator of adipogenesis; *IRS-1* and *IRS-2*; *KCNJ11*, which encodes a component of the potassium ATP channel in pancreatic β cells; *WFS-1*, which is involved in the stress response of the endoplasmic reticulum in β cells; and *HNF1A, HNF1B* and *HNF4A*, which encode hepatocyte nuclear factors (HNFs) implicated in glucose homeostasis [96–105]*.* A family history of diabetes is also a significant risk factor, as having one parent with T2DM results in a 40% chance of their child having the disease, and if both parents have T2DM, the chance is approximately 70% [106,107]. There is also a risk of developing T2DM if one's mother had diabetes while pregnant [108]. How exactly these genes interact with each other, other genes, and environmental factors to result in T2DM is an area of ongoing study.

### 5.3. Risk factors for GDM

Like T2DM, a poor diet, obesity, a sedentary lifestyle, smoking, family history of T2DM, and a mother who had GDM makes a woman more likely to develop GDM [109–111]. Studies have shown conflicting statistical probability in paternal diabetes, that is, a father being diabetic, being a risk factor for a woman developing GDM [112,113]. Unique risk factors for developing GDM are being pregnant when greater than or equal to 40 years old, carrying a male fetus, suffering from depression during the first and second trimesters, and higher exposure to organic pollutants and endocrine disruptors [109,114–117]. There is potentially a greater risk of developing T2DM if the mother is non-White, though non-White populations are at greater risk of experiencing the lifestyle factors known to contribute to T2DM [109].

Genes that may be associated with GDM have been difficult to establish and have not always been consistent across studies. A recent meta-analysis found that single nucleotide polymorphisms (SNPs) in *TCF7L2, KCNJ11* and *IRS-1* have a significant association with GDM [118]. Other SNPs found to increase risk uniquely in GDM are *GCK*, encoding glucokinase; *CDKAL1*; *IGF2BP2,* encoding insulin-like growth factor 2 mRNA-binding protein 2*;* and *MTNR1B*. All four of these genes are involved in insulin secretion [118]. However, 17 of the 29 studies pooled for that analysis included only White women and, in the consequent analysis, not all ethnicities were affected the same by the SNPs [118]. There is a great need for more GDM-associated genetic studies in non-White populations.

## 6. Current methods of studying diabetes

Animal models remain the best way of studying the various types of diabetes due to the complexity of the disease, which non-animal models cannot always replicate. The most commonly used animals are mice and rats, although strains vary in diabetic predisposition. Choosing the correct strain is crucial.

Importantly, females of some rodent strains used as diabetic models have decreased reproductive ability, such as Zucker rats, Zucker diabetic fatty rats, Goto-Kakizaki (GK) rats, Otsuka Long-Evans Tokushima Fat (OLETF) rats, Lep^db/db^ mice, Lep^ob/ob^ mice and New Zealand obese (NZO) mice [119–125].

Chemical, dietary, genetic and viral models exist for both species to study T1DM, T2DM and GDM. To model maternal diabetes, a female rodent should undergo the desired method before being set with her mate; the methods for T1DM and T2DM outlined below suffice to accomplish this.

### 6.1. Chemicals

#### 6.1.1. Streptozotocin

**Figure 3.**
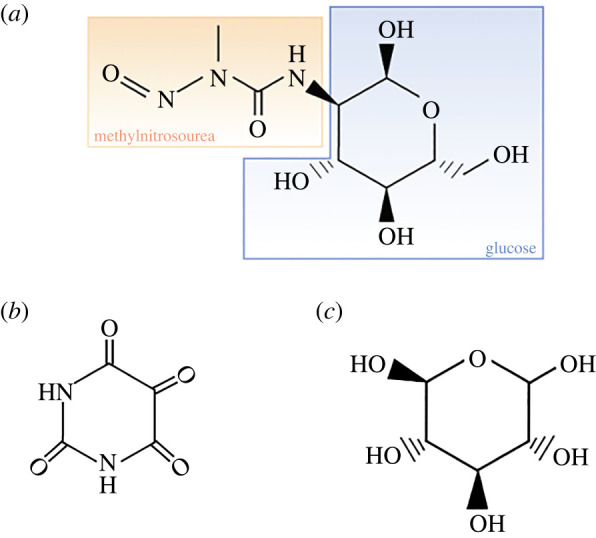
Chemical structures of chemicals used in diabetes modelling. (*a*) Structure of streptozotocin, with methylnitrosourea and glucose moieties labelled in orange and blue, respectively. The methylnitrosurea moiety in streptozotocin makes it cytotoxic, whereas the glucose moiety allows it to be transported into the β cell by GLUT2, (*b*) structure of alloxan and (*c*) structure of glucose.

Streptozotocin (STZ) is an antibiotic derived from *Streptomyces achromogenes* [126]. Its administration can be intraperitoneal or intravenous at a single high dose or multiple low doses, with doses adjusted to the animal's weight [127]. As noted in figure 3, STZ is composed of a glucose moiety that binds to the glucose transporter 2 (GLUT2) receptor on the β cell membrane and a methylnitrosourea moiety that lends it cytotoxicity by alkylating DNA [128,129]. STZ ultimately causes hyperglycaemia by reducing ATP, thereby preventing insulin secretion [130]. Hyperglycaemia further impairs the GLUT2-possessing liver and kidneys from functioning, mimicking organ complications in human diabetes [128]. Pancreatic islet destruction due to the competition between STZ and glucose is suitable for modelling T1DM. T2DM can also be modelled with dietary modifications, the concomitant use of nicotinamide, or neonatal injections [127,128]. STZ-induced diabetes can last up to three months [131]. However, mortality can be high due to STZ targeting several organs. One rat study reported a 20% mortality rate [132]. For older rats, the rates are even higher: 83% for 12 to 17-week-old rats and 91% for rats greater than or equal to 18 weeks old [133].

However, not all rodent strains are equally susceptible to STZ-induced diabetes. Female C57Bl/6 J mice are resistant to STZ; however, previous studies have increased dosage to successfully induce diabetes [134–137]. Female Zucker diabetic fatty rats and Balb cJ^−1^ mice also exhibit STZ resistance [138,139].

#### 6.1.2. Alloxan

Similar to STZ, alloxan is a glucose competitor that binds to GLUT2, but alloxan is cytotoxic due to the production of reactive oxygen species [129]. Alloxan can also affect the liver and kidneys [129]. Its administration can be in one high dose or multiple low doses adjusted by weight. However, unlike STZ, alloxan can be administered subcutaneously [131]. It has a high mortality rate in mice, 30–60%, likely due to its generation of reactive oxygen species [131]. Alloxan is better suited for modelling T1DM than T2DM [131]. Although less expensive per gram than STZ, alloxan is less commonly used, partly due to having multiple blood glucose response phases [131]. This makes the resulting hyperglycaemia less stable but, even if stable hyperglycaemia is achieved, its average duration is just 1 month [131]. Given that mouse and rat gestation periods also last roughly one month and that alloxan has a variable blood glucose response, we do not recommend the use of alloxan to model maternal diabetes. Like STZ, females of rodent strains react differently to alloxan. Albino female rats have higher susceptibility to alloxan-induced diabetes than males [140].

### 6.2. Dietary

Obesity is a T2DM risk factor and, in mice, a diet high in fat and simple carbohydrates reproduces obesity as well as T2DM [141]. Because islet destruction is not induced as in T1DM, this model is only suitable for modelling T2DM. A high-fat diet is typically used in conjunction with other models of diabetes induction, such as STZ or strains of animals genetically predisposed to obesity, such as Lep^ob/ob^ mice [127,142]. These multifactorial models more faithfully replicate the pathogenesis of T2DM in humans [143].

However, in females of some strains, such as C57Bl/6, mice are resistant to glucose intolerance, hyperinsulinaemia, and insulin resistance induced by a high-fat diet [144–146]. Further, young BALB/c females are resistant to obesity caused by a high-fat diet, although this changes as they age [147].

### 6.3. Genetic

Several strains of mice and rats have been developed to address the different etiologies of diabetes. Strains exist that model β cell destruction as seen in both T1DM and T2DM, with the strains differing in the reason for their destruction, and models for obesity as predisposed by T2DM [127].

Non-obese diabetic (NOD) mice, diabetes-prone BioBreeding rats, and LEW.1AR1/-iddm rats are strains of rodents that develop diabetes spontaneously due to autoimmune processes and without an obesity factor, making them ideal for T1DM studies [148–150]. GK rats are the most commonly used non-obese non-insulin-dependent diabetes animal model, though β cell mass and the extent of metabolic dysfunction differs between colonies, and females have lower blood sugar levels than males [151–153]. By contrast, AKITA mice exhibit diabetes and its complications not from autoimmunity, but from stress on the endoplasmic reticulum of β cells caused by misfolded insulin [154,155]. While AKITA mice have been used for T2DM studies, they are best used for T1DM, as their diabetic development is still spontaneous. Importantly, NOD female mice exhibit a higher incidence of T1DM than males [156].

Because obesity is a major risk factor for T2DM, several strains of obesity-prone rodents have been developed. For monogenic studies of diabetes, leptin-deficient Lep^ob/ob^ mice or leptin receptor-deficient Lep^db/db^ mice, Zucker fatty rats, and Zucker diabetic fatty rats are used [127]. For polygenic studies, KK mice, OLETF rats, NZO mice, TallyHo/Jng mice and NoncNZO10/LtJ mice are used [127]. However, OLETF female rats have a markedly lower incidence of diabetes than males, and they do not suffer from renal complications [157].

Transgenic mouse models of T2DM also exist. hIAPP transgenic mice express the human transgene under the rat II insulin promoter. In this mouse model, hIAPP aggregates cause β cell death, which is an underlying pathological hallmark of T2DM [158]. The human form is used because rodent IAPP does not inherently form these aggregates [159]. However, hIAPP females have a much lower incidence of diabetes compared to males, occurring in around 11% of female hIAPP mice but greater than 80% of males [60].

### 6.4. Viral

Viruses have been proposed to lend susceptibility to T1DM in humans [10]. Rodent models of virally induced diabetes, caused by the virus either inducing autoimmunity or directly infecting β cells, have been created using Coxsackie B virus, encephalomyocarditis virus, Killham rat virus and lymphocytic choriomeningitis virus under the rat insulin promoter [10,127].

## 7. Relevance to maternal and fetal health

Defects in babies born to diabetic mothers, termed diabetic embryopathies, are known to occur with both T1DM and T2DM [160]. These include anorectal atresia/stenosis, caudal dysgenesis, congenital heart defects, costovertebral segmentation defects, holoprosencephaly, longitudinal birth defects, microtia/anotia/hemifacial microsomia, neural tube defects, renal aplasia and dysplasia, sirenomelia, thymus aplasia and urorectal septum malformations [161–172]. Other embryopathies that have been reported include bifid tongue, cleft lip/palate, facial dysmorphism, hydrocephaly, congenital hypertrophic cardiopathy and septo-optic dysplasia [161,173–179]. Spontaneous abortions are also possible [180]. Pregnancy loss rates are similar for both T1DM and T2DM-affected pregnant mothers, but they differ in their causes [181]. Mothers with T1DM tend to lose their fetus from embryopathies and neonatal prematurity complications linked to lack of glycemic control during early pregnancy [181–183]. Conversely, mothers with T2DM are more likely to lose their fetus because of stillbirth, birth asphyxia and chorioamnionitis [181]. Maternal obesity and hyperglycaemia are risk factors for chorioamnionitis, as is poverty, which correlates with obesity [181,184–186].

Children born to mothers with T1DM are at risk of being born via Caesarean section, increased perinatal mortality, congenital anomalies, abnormal gestational size, shoulder dystocia, hypoglycaemia (low blood glucose levels), polycythaemia (increased red blood cell mass), hypocalcaemia (low levels of calcium in the blood), respiratory distress syndrome, perinatal mortality and fetal death [11,160,187]. As children born to mothers with T1DM age, they are more likely to become obese, have glucose intolerance, and develop T1DM or cardiovascular disease [12,13]. Children born to mothers with T2DM are likewise at risk of congenital deformities, hypoglycaemia, later development of T2DM, perinatal mortality and fetal death [14,160,188,189].

Obesity is a major risk factor for T2DM, and it is more prevalent in women than in men [190]. Moreover, maternal obesity can cause complications in the child, such as birth by Caesarean section, having a high birth weight contributing in part to the child's own vulnerability to obesity, too low of a birth weight, neural tube defects, heart defects, oral clefts, skeletal anomalies, hypoglycaemia, hyperinsulinaemia and fetal death [191–196]. Long term, the child is more likely to suffer from obesity [197].

Children born to GDM-affected mothers may be large for their gestational age, to have shoulder dystocia, hypoglycaemia, hypocalcaemia and hyperbilirubinaemia [198]. Later in life, they can become obese and suffer from glucose intolerance and metabolic syndromes, including the development of T2DM itself [197–199].

Because what is diagnosed as GDM may in fact be early onset T2DM, whether GDM causes congenital birth defects is controversial. Some studies have found that the difference between GDM-affected mothers and non-diabetic mothers in having children with birth defects is not significant, while others have, the latter particularly when considering women who may have had yet-undiagnosed T2DM [174,200,201].

**Figure 4.**
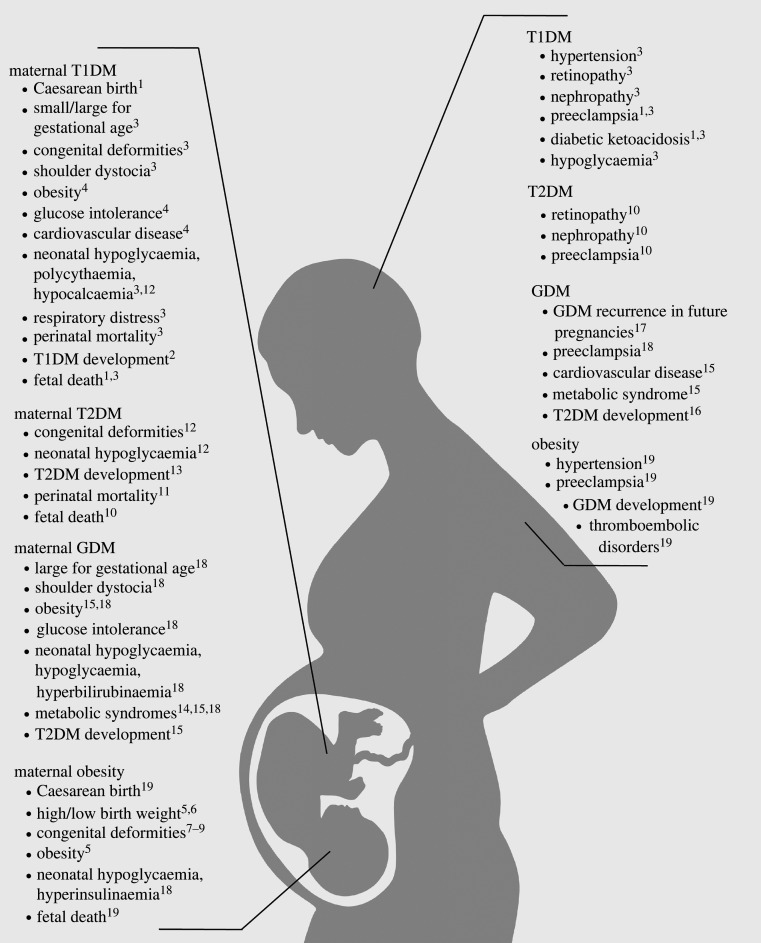
Overview of fetal (left) and maternal (right) complications from T1DM, T2DM, GDM and obesity, a T2DM risk factor. Citations are provided in the electronic supplementary material.

Adverse pregnancy outcomes affect not only the health of the baby, but that of the mother (figure 4). Compared to non-diabetic mothers, women with T1DM who become pregnant are far more likely to suffer from hypertension; retinopathy; nephropathy; preeclampsia; diabetic ketoacidosis; and hypoglycaemia, which severely affects 45% of T1DM-affected pregnant women, particularly early in the pregnancy [11,187]. Maternal complications of T2DM also include retinopathy, nephropathy, and preeclampsia [188]. Although GDM resolves in approximately 90% of affected pregnancies, it is likely to recur in future pregnancies [202]. Furthermore, a GDM-affected woman is at risk of later developing T2DM [203]. Cardiovascular disease and metabolic syndrome are also common among women who have had GDM, and both of these diseases have risk factors much like those seen in diabetes: obesity, hypertension, insulin resistance and dyslipidaemia [199]. Moreover, metabolic syndrome can contribute to the onset of diabetes and cardiovascular disease [199].

As of 2016, 0.9% of pregnancies in the USA occurred in women with maternal diabetes. Notably, the percentage of maternal diabetes has remained stable from previous years, but the prevalence of GDM has been increasing [204]. Due to the lack of consistent diagnostic criteria for GDM, the exact number of GDM pregnancies may vary by measure, but it is undeniable that the prevalence of such pregnancies is increasing globally [199]. This is related in part to the similar worldwide increase in obesity, a risk factor for T2DM and GDM [199].

GDM is preferably treated by a change in nutrition, although physical activity, pharmacological agents and blood glucose monitoring can also help [205]. The most common pharmacologic of choice is insulin, though metformin and glyburide are also approved for use in pregnant women [24,205,206]. Though no birth defects have been noted in babies born to women taking any of these three drugs, long-term changes in the metabolism of the children have not been well studied [206]. Studies comparing the treatment of T2DM-affected and GDM-affected pregnant women with metformin versus insulin have reported mixed results: some have found metformin to be superior in achieving normoglycaemia and reducing hypoglycaemic episodes, whereas others have found maternal/fetal outcomes to be similar between treatment with the two drugs [207,208]. In simply treating GDM-affected women, however, a meta-analysis concluded that metformin is better than insulin in reducing weight gain and hypertension, and in maintaining normoglycaemia, though fetal outcomes were similar for both drugs [209]. Somewhat worryingly, metformin can cross the placenta and result in fetal levels of metformin nearly equal to that of the mother [210]. Metformin-exposed neonates can be smaller at birth, but follow-up studies have found that, by 9 years old, the children tend to be larger than insulin-exposed children, with higher BMI [211,212]. The lag in their growth puts them at higher risk of developing cardiac and metabolic diseases [211]. However, neurodevelopment is similar between children whose mothers were treated with metformin and mothers treated with insulin [213]. Other long-term effects of metformin in offspring are unknown [206]. A meta-analysis comparing GDM-affected women treated with glyburide versus insulin did not find differences in adverse perinatal outcomes, but a more recent meta-analysis found that neonates exposed *in utero* to glyburide have a higher incidence of hypoglycaemia [214,215]. Few studies have compared metformin to glyburide, but in those that have, GDM-affected women treated with glyburide had greater weight gain and had babies with higher birth weight, macrosomia and large for their gestational age [216].

## 8. Future directions and unanswered questions

An issue with most diabetes research in mice is that males are overwhelmingly used, but as established, there are sex differences in the severity of disease complications in rodents, just as there are in humans [217]. Likewise, in humans, the incidence of diabetes in women across most age groups is lower than that of men [4]. Why this is so has been attributed to the protective role of ooestrogen, as ooestrogen deficiency predisposes to risk factors of diabetes and T2DM itself; further, following menopause, incidence of diabetes in women rises [4,218]. In the context of maternal diabetes, only female mice can be studied, making it important to separate expectations between the sexes.

T2DM and GDM are multifactorial diseases, with modifiable lifestyle factors playing the greatest role in disease manifestation. Prevention of T2DM and GDM by a healthy lifestyle and diet remains the best approach, but certain genes—particularly involved in insulin secretion, glucose homeostasis and adipogenesis—have been identified as risk factors [96–105,118]. Conversely, T1DM arises from autoimmune dysfunction, with mutations in *HLA* posing significant genetic predisposition to its development [78]. Clinical onset of T2DM and T1DM is not observed until there has been a significant loss of β cell mass and/or function, thus pharmaceuticals that would prevent this would prove the most beneficial. Cell death in T2DM is caused by hyperglycaemia and hyperlipidaemia while in T1DM, it is by the body's own immune system; it remains unknown how exactly these conditions induce apoptosis, but stress in the endoplasmic reticulum has been implicated in both T1DM and T2DM [61,219].

Despite identified diabetic predisposition genes, they have not always been found across diverse populations. There has also been difficulty in discerning the molecular specifics by which these genes alter the probability of getting diabetes, and in uncoupling the association of lifestyle factors from the studies. SNPs in *TCF7L2* have been found to be strongly associated with the development of GDM, just as they are in T2DM [220]. The trend of T2DM risk holds true even across different races and ethnicities [221]. However, how it contributes to diabetic risk is not well understood. *TCF7L2* is part of the Wnt signalling pathway, which is involved in multiple developmental processes, including adipogenesis [221]. The role of *TCF7L2* and Wnt in the regulation of adipogenesis is an area of ongoing study. *KCNJ11* encodes ATP-sensitive potassium channel proteins found in pancreatic β cells [222]. SNPs in *KCNJ11* have also been identified as lending susceptibility to T2DM [222]. Mutations in *KCNJ11* inhibit the ability of ATP to regulate the potassium channel while enhancing stimulatory magnesium, altering insulin secretion and ultimately causing diabetes [222]. Exactly how this occurs remains to be understood. Furthermore, not all SNPs in *KCNJ11* have been specifically associated with altered risk in all types of diabetes [222]. More investigation is needed to fully understand how different SNPs impact function in diabetes. *GCK* encodes glucokinase, an enzyme critical for glycolysis and insulin regulation that is expressed in pancreatic β cells [223]. It has been identified as a GDM risk factor in White women, but at least one study did not find it to be the case in North Indian women [224]. Recently, *MTNR1B* has been implicated in several diverse populations to affect β cell activity by reducing insulin secretion [36,225]. The specifics of how this can potentially result in diabetes remain to be studied. *CDKAL1* has also been identified as a gene that increases diabetic onset in White and non-White populations [36]. Though it has mostly been studied for its downstream effects on the translation of insulin, obesity has also been found to downregulate its mRNA levels in mouse adipose tissue, as *CDKAL1* loss affects adipose mitochondrial function [226]. How this is accomplished is unclear.

Though there are a few approved drugs for use in diabetic pregnant women, more long-term follow up studies in their children are needed, particularly for those drugs that can cross the placenta. Because ooestrogen has shown to have a protective effect against diabetes, ooestrogen receptors are an attractive potential pharmaceutical target [227]. However, sex differences in metabolic regulation have not been fully characterized, and the inherent hormonal and metabolic changes in pregnancy, coupled with diabetes pose additional challenges.

Because the yolk sac and later the placenta are the sites of maternal–fetal nutrient exchange, and the diet of the mother can influence the availability of nutrients for the developing fetus, genes involved in glucose and fatty acid metabolism—as affected in diabetes—that are active in the yolk sac and placenta are of primary interest as potential therapeutic targets [228,229]. In rats, hyperglycaemia induces vascularization defects in the yolk sac, concomitant with embryopathies in the embryo [230,231]. The content of almost all types of fatty acids in yolk sacs from hyperglycaemic rats is higher than the amount found in embryos [232]. Conversely, the morphology of placentas from diabetic mothers do not differ from placentas from non-diabetic mothers, but they do histopathologically [233–235]. Rat embryos with *in vitro* or *in vivo* addition of arachidonic acid, a long-chain fatty acid, or with myo-inositol, a sugar alcohol, reduce the incidence of neural tube defects [232,236,237]. Arachidonic acid is created from precursor fatty acids, and in diabetic rats, this process is defective; human studies have reported the same [238,239]. Myo-inositol, which has insulin-like properties, improves glucose uptake and inhibits lipolysis in treated cells, and reduces insulin resistance in women with GDM [240,241]. How arachidonic acid and myo-inositol supplementation achieve these embryopathy-reducing effects in a hyperglycaemic environment is unclear, but because arachidonic acid is enriched in phosphatidylinositol, and phospholipid metabolism by PI3-K is requisite in insulin signalling as is the inositol phosphoglycan pathway, it could be that insufficient levels of arachidonic acid and inositol in diabetics affect insulin signalling [242–244].

In the last few decades, there has been increased interest in novel therapeutics, such as antibody treatment and gene therapy. Antibody treatment has yielded promising results in humans. In T1DM patients, anti-CD3 therapy has been shown to reverse hyperglycaemia and improve insulin production up to a year after injection; anti-C20 therapy has been shown to delay β cell degradation, though it does not halt the disease; and anti-CD2 therapy has been shown to improve β cell function, even a year after therapy ended [245–247]. The challenge has been in maintaining the positive effects long-term and eradicating the root cause of the disease. For T2DM, antibodies against amyloid polypeptide have been developed, blocking aggregate formation and reducing T2DM symptoms; however, this has only been tested in mice [248]. None of these antibodies have been tested in pregnant women, but ethical concerns arise in such possible studies. Gene therapy for diabetes has successfully reversed the obese and consequent diabetic phenotype in Lep^ob/ob^ mice, and in NOD mice, gene therapy has been shown to return blood glucose to normal levels in 80% of mice [249,250]. Trials of human gene transfer to treat T1DM have been approved in humans [251].

## 9. Conclusion

Environmental, lifestyle and genetic factors contribute to diabetes mellitus, a metabolic disease affecting insulin production and usage that can be further categorized by the molecular characterization of disease onset. T1DM is an autoimmune disease caused by the body destroying its own insulin-producing pancreatic β cells [10]. T2DM is typically acquired due to poor diet and subsequent insulin resistance, leading to dangerous increases in blood glucose, ultimately killing pancreatic β cells [51]. GDM can be acquired during pregnancy due to natural changes in maternal metabolism, but T2DM can then develop in the mother after pregnancy [36]. Regardless of the type of diabetes a pregnant woman has, whether onset during pregnancy or present prior to it, adverse health outcomes in her and her child are more likely to occur compared to non-diabetic mothers, and a baby born to a diabetic mother is more likely to develop diabetes [11,191,199]. With the global rise in diabetes and the high costs of diabetic pharmaceuticals, both health and economic crises loom.

Mice and rats are the most common animal models of diabetes. There are chemical, dietary, genetic and viral methods of studying diabetes, each with varying efficiency and analogy to disease progression in humans. When not focused on GDM or maternal diabetes, most of these studies use male rodents, proving problematic when applied to female rodents because of sex differences in disease severity and consequences in both rodents and humans [217]. Furthermore, several strains of female rodents, or female rodents under chemical or dietary treatment, exhibit resistance to developing diabetes [60,137–140,144–146,153,157]. Reproductive ability in some rodent strains used for diabetic studies can also be affected [119–125].

In humans, lifestyle factors contributing to T2DM and GDM have been clearly established [91,109,110]. Ongoing studies are attempting to discern the roles of identified genes, such as *TCF7L2, KCNJ11, GCK, MTNR1B* and *CDKAL1*, most of which are involved in adipogenesis, insulin secretion or glucose metabolism [221–223,225,226]. However, there is a great need to replicate these studies in non-White populations, particularly because the prevalence of diabetes is increasing more in non-Western countries [94]. Though there are drugs that can be used to treat diabetes during pregnancy, the safety and efficacy of most oral medications remain to be elucidated in this population. Of the diabetes medications that are currently used, the long-term effects on the child are not well known [24,206]. Diabetic antibody therapeutics are a rather new treatment with good short-term outcomes, but they have not been tested in pregnant women [245–247]. Diabetic gene therapy is another potential treatment, though clinical trials remain to establish their safety and efficacy in people, much less in pregnant women. Because diabetes in pregnancy puts the child at risk of diabetes development, finding safer, more cost-effective treatment options that target genes or molecular etiologies across ethnically diverse pregnant women can help halt the recursive, increasing trend of diabetes.

## Data Availability

The data are provided in electronic supplementary material [252].
